# 11-Oxygenated Estrogens Are a Novel Class of Human Estrogens but Do not Contribute to the Circulating Estrogen Pool

**DOI:** 10.1210/endocr/bqaa231

**Published:** 2020-12-19

**Authors:** Lise Barnard, Lina Schiffer, Renate Louw du-Toit, Jennifer A Tamblyn, Shiuan Chen, Donita Africander, Wiebke Arlt, Paul A Foster, Karl-Heinz Storbeck

**Affiliations:** 1 Department of Biochemistry, Stellenbosch University, Matieland, South Africa; 2 Institute of Metabolism and Systems Research (IMSR), College of Medical and Dental Sciences, University of Birmingham, Birmingham, UK; 3 Centre for Women’s & Newborn Health, Birmingham Health Partners, Birmingham Women’s & Children’s NHS Foundation Trust, Birmingham, UK; 4 Centre for Endocrinology, Diabetes and Metabolism, Birmingham Health Partners, University Hospitals Birmingham NHS Foundation Trust, Birmingham, UK; 5 Department of Cancer Biology, Beckman Research Institute, Duarte, California USA; 6 National Institute for Health Research (NIHR) Birmingham Biomedical Research Centre, University Hospitals Birmingham NHS Foundation Trust and University of Birmingham, Birmingham, UK

**Keywords:** 11-oxygenated androgens, 11-oxygenated estrogens, cytochrome P450 aromatase, 11-ketotestosterone, 11-ketoestradiol, 11-ketoestrone

## Abstract

Androgens are the obligatory precursors of estrogens. In humans, classic androgen biosynthesis yields testosterone, thought to represent the predominant circulating active androgen both in men and women. However, recent work has shown that 11-ketotestosterone, derived from the newly described 11-oxygenated androgen biosynthesis pathway, makes a substantial contribution to the active androgen pool in women. Considering that classic androgens are the obligatory substrates for estrogen biosynthesis catalyzed by cytochrome P450 aromatase, we hypothesized that 11-oxygenated androgens are aromatizable. Here we use steroid analysis by tandem mass spectrometry to demonstrate that human aromatase generates 11-oxygenated estrogens from 11-oxygenated androgens in 3 different cell-based aromatase expression systems and in human ex vivo placenta explant cultures. We also show that 11-oxygenated estrogens are generated as a byproduct of the aromatization of classic androgens. We show that 11β-hydroxy-17β-estradiol binds and activates estrogen receptors α and β and that 11β-hydroxy-17β-estradiol and the classic androgen pathway-derived active estrogen, 17β-estradiol, are equipotent in stimulating breast cancer cell line proliferation and expression of estrogen-responsive genes. 11-oxygenated estrogens were, however, not detectable in serum from individuals with high aromatase levels (pregnant women) and elevated 11-oxygenated androgen levels (patients with congenital adrenal hyperplasia or adrenocortical carcinoma). Our data show that while 11-oxygenated androgens are aromatizable in vitro and ex vivo, the resulting 11-oxygenated estrogens are not detectable in circulation, suggesting that 11-oxygenated androgens function primarily as androgens in vivo.

In humans, androstenedione (A4) is the immediate substrate for testosterone biosynthesis. Testosterone (T) and its 5α-reduced form, 5α-dihydrotestosterone (DHT), are potent human androgens that bind and activate the human androgen receptor (AR). In the human adrenal, A4 is also converted to 11β-hydroxyandrostenedione (11OHA4), previously thought to be an inactive, dead-end metabolite. However, recent studies have shown that this abundant adrenal steroid serves as the key precursor for the newly described 11-oxygenated androgen biosynthesis pathway, through consecutive conversion to 11-ketoandrostenedione (11KA4) and 11-ketotestosterone (11KT) (1-5). 11KT is a potent androgen that binds and activates the AR with similar affinity, potency, and efficacy to T (1, 6). Subsequent studies have shown that 11KT circulates at similar or higher levels than T in healthy women and represents the predominant circulating androgen in androgen-excess conditions such as polycystic ovary syndrome and congenital adrenal hyperplasia (7-10). Moreover, unlike classic androgens, circulating levels of 11-oxygenated androgens appear not to decline after menopause (8, 11).

T serves as a precursor to the potent estrogen 17β-estradiol (E_2_), while A4 can be converted to the weaker estrogen estrone (E_1_). Since androgens are obligatory precursors to estrogen biosynthesis catalyzed by the enzyme cytochrome P450 aromatase (CYP19A1), it appears eminently plausible that 11-oxygenated androgens could also serve as substrates for aromatase, potentially yielding a novel class of active, 11-oxygenated estrogens. Here we comprehensively investigated the biosynthesis and estrogenic activity of 11-oxygenated estrogens.

## Materials and Methods

### Cell lines

HEK293, COS-1, and JEG3 cells were purchased from the American Type Culture Collective. MCF7 cells were purchased from the European Collection of Authenticated Cell Cultures, and MCF7-BUS cells were a gift from A. Soto. HEK293 and MCF7-BUS cells were cultured in Dulbecco’s Modified Eagle Medium (DMEM) supplemented with 10% fetal bovine serum (FBS) (heat-inactivated for MCF7-BUS cells) and 1% penicillin-streptomycin, while JEG3, MCF7, and MCF7arom were maintained in Eagle’s Minimum Essential Medium supplemented with 10% FBS and 1% penicillin-streptomycin. MCF7arom cells were developed and maintained as previously described (12). Cell lines were authenticated by short-tandem repeat profiling (NorthGene) and were regularly tested for mycoplasma contamination.

### Plasmid constructs

The pcDNA4/17β-hydroxysteroid dehydrogenase type 2 (HSD17B2) and pcDNA4/HSD17B4 plasmids were gifts from J. Adamski. The pCR3/11β-hydroxysteroid dehydrogenase type 2 (HSD11B2) plasmid was a gift from P.M. Stewart. W.L. Miller provided the pCMV/bovine cytochrome P450 11β-hydroxylase (CYP11B1) and pCMV/CYP11B2 plasmids, and the pCIneo/ADX plasmid were from R.C. Tuckey. The pcDNA3.1/aromatase plasmid was purchased from Genscript Biotech. The pSG5/hERα and pSG5/hERβ plasmid vectors were from F. Gannon, and the pGL3-2xERE-pS2-luciferase promoter reporter construct was from B. Belandia.

### Steroids

11β-hydroxyandrostenedione (4-androsten-11β-ol-3,17-dione; 11OHA4), 11β-hydroxy-17β-estradiol (1,3,5(10)-estratrien-3,11β,17β-triol; 11OHE_2_), 11β-hydroxytestosterone (4-androsten-11β,17β-diol-3-one; 11OHT), 11-ketoandrostenedione (4-androsten-3,11-17-trione; 11KA4), 11-ketotestosterone (4-androsten-17β-ol-3,11-dione; 11KT), androstenedione (4-androsten-3,17-dione; A4) and T (4-androsten-17β-ol-3-one; T) were purchased from Steraloids. 17β-Estradiol (1,3,5(10)-estratrien-3, 17β-diol; E_2_), bisphenol A (4,4′-(propane-2,2,diyl)diphenol; BPA), estrone (1,3,5(10)-estratien-3-ol-17-one; E_1_), fulvestrant (ICI), and letrozole were purchased from Sigma-Aldrich, and 11β-hydroxyandrostenedione 2,2,4,6,6,16,16-D7 (D7- 11OHA4) and T 1,2-D2 (D2-T) were from Cambridge Isotopes. [2,4,6,7-^3^H(N)]-estradiol ([^3^H]-E_2_) and [1β-^3^H(N)]-androst-4-ene-3,17-dione ([^3^H]-A4) were from PerkinElmer Life.

### Steroid conversion by CypExpress Aromatase

Aromatase conversion by the CypExpress Aromatase eukaryotic system (Oxford Biomedical Research) was carried out according to the manufacturer’s protocol. Each reaction contained 20-mg CypExpress powder, 5-mM glucose-6-phosphate, 2-mM oxidized nicotinamide adenine dinucleotide phosphate (NADP^+^) (Roche Diagnostics), and 1-µM steroid substrate (with or without 10-µM letrozole) in 1-mL 100 mM potassium phosphate buffer (pH 7.4). Reactions were incubated at 37 °C for 4 hours with continuous stirring.

### Aromatase assays in aromatase-expressing cell lines

Aromatase activity in the MCF7, MCF7arom, and JEG3 cells were compared using a tritiated water assay as previously reported (12). Subsequently the MCF7arom and JEG3 cells were treated with 1 µM of the appropriate steroid (A4, T, 11OHA4, 11OHT, 11KA4, 11KT with and without 10-µM letrozole) for 24 hours. The protein concentration of the cell lysate was determined using a Pierce BCA kit (Thermo Fisher Scientific).

### Enzyme assays in transiently transfected HEK293 cells

CYP11B1 and CYP11B2 enzyme assays were performed in transiently transfected HEK293 cells as previously described (13).

### Steroid conversion in placenta tissue

Fresh human placental tissue was obtained from healthy pregnant women undergoing elective cesarean delivery at term (37-40 weeks’ gestational age; as determined by ultrasound measurement of crown rump length) at the Birmingham Women’s Hospital, Birmingham Women’s & Children’s Hospital Foundation Trust, Birmingham, UK. All participating women provided written informed consent prior to tissue collection (13/WM/0178 [2013]). Placental samples were identified macroscopically under sterile conditions and washed thoroughly with phosphate-buffered saline. Full-thickness, whole-tissue biopsies (decidual and trophoblast) (500-1000 mg) were dissected, washed in phosphate-buffered saline, and dissected into 4 to 6 pieces and added to 5 mL phenol-red free DMEM/F12 media containing 1% penicillin-streptomycin and 1-µM steroid substrate. Placental explants were incubated for 40 hours at 37 °C in a hybridizing oven.

### Human serum

Maternal serum and serum from the umbilical cord were obtained from healthy pregnant women undergoing elective cesarean delivery at term (37-40 weeks’ gestational age; as determined by ultrasound measurement of crown rump length) at the Birmingham Women’s Hospital, Birmingham Women’s & Children’s Hospital Foundation Trust. All participating women provided written informed consent prior to tissue collection (13/WM/0178 [2013]). In addition, we analyzed serum samples from patients with congenital adrenal hyperplasia due to 21-hydroxylase deficiency and patients with adrenocortical carcinoma in situ, selected for high-circulating 11-oxygenated androgen concentrations and recruited from the Adrenal Clinic at the Queen Elizabeth Hospital Birmingham; patients had provided written informed consent prior to serum collection (University of Birmingham Human Biomaterials Resource Centre ethics application approved by North West 5 Research Ethics Committee, Haydock Park; reference No. REC 09/H1010/75).

### Steroid extraction and derivatization

Steroids were extracted from in vitro samples using methyl tert-butyl ether (MTBE) as previously described (13, 14). D2-T (1.5 ng), D7-11OHA4 (15 ng), and BPA (15 ng) were used as internal standards. MTBE was replaced with ethyl acetate (2.5 mL) for the extraction of estrogens from 500-µL serum. Estrogens were derivatized using dansyl chloride (15-17).

### Steroid analysis by ultra-high performance liquid chromatography tandem mass spectrometry

Steroids were separated and quantified using an ACQUITY UHPLC (Waters Corp) coupled to a Xevo-QTS triple quadrupole mass spectrometer (Waters Corp). Chromatographic separation was achieved using a BEH C18 (2.1 mm × 50 mm; 1.7 µM) column (Waters Corp) at 60 °C. The mobile phases consisted of 1% formic acid (solvent A) and 1% formic acid in methanol (solvent B) at a constant flow rate of 0.6 mL/min. Separation was achieved using an initial isocratic period (0.5 minutes) at 40% B; a 1.5-minute linear gradient from 40% B to 60% B; a 0.5-minute linear gradient from 60% B to 75% B; and a 1.5-minute linear gradient from 75% B to 85% B. The injection volume was 5 µL. Steroids were detected and quantified using multiple reaction monitoring (MRM) in positive electrospray ionization mode as shown in Table 1. In the absence of commercial standards, CypExpress (aromatase) was used to biosynthesize 11β-hydroxyestrone (11OHE_1_), 11-ketoestrone (11KE_1_), and 11-keto-17β-estradiol (11KE_2_) from 11OHA4, 11KA4, and 11KT, respectively. We are confident of the correct identification of 11-oxygenated estrogens from their 11-oxygenated androgen precursors because: (1) all biosynthesis was inhibited by the aromatase-specific inhibitor, letrozole; (2) dansyl chloride, an estrogen-specific derivatization agent, was used to successfully derivatize the products; and (3) the masses of the dansyl chloride–derivatized products correspond to the predicted 11-oxygenated estrogen masses when derivatized by dansyl chloride. The mass spectrometer instrumental parameters were as follows: source temperature, 150 °C; desolvation temperature, 350 °C; desolvation gas flow, 800 L/hour; capillary voltage, 3.7 kV; cone voltage, 30 V; collision energy, 15 to 45 electron volt (eV); cone gas flow, 150 L/hour; and collision gas flow, 0.15 mL/minute.

**Table 1.** Multiple reaction monitoring parameters for steroid analysis

Steroid metabolite	RT, min	Molecular ion, m/z	CV, V	Quantifier ion, m/z	CE, eV	Qualifier ion, m/z	CE, eV
A4	2.02	287.2	30	96.9	15	108.8	15
T	2.19	289.2	30	97.2	22	109	22
11OHA4	1.50	303.2	30	267.2	15	121	30
11KA4	1.22	301.2	35	257	25	265.2	25
11OHT	1.64	305.2	35	269	15	121	20
11KT	1.38	303.2	30	121	20	267	18
E_1_	3.53	504	20	171	35	156	45
E_2_	3.62	506	35	171	35	156	40
11OHE_1_	3.00	520.2	15	171	35	170	35
11KE_1_	2.98	518.2	15	171	35	170	35
11OHE_2_	3.16	522.2	15	171	35	504.2	20
11KE_2_	3.06	520.2	15	171	35	170	35
*D2-T*	2.19	291	30	99.1	20	111.2	30
*D7-11OHA4*	1.49	310.2	25	147.2	25	99.8	30
*BPA*	4.40	695	50	171	35	170	35

Retention time, molecular ion species, MRM mass transitions, CV, and CE for each steroid are reported. Internal standards: D2-T, D7-11OHA4, and BPA.

Abbreviations: 11KA4, 11-ketoandrostenedione; 11KE_1_, 11-ketoestrone; 11KE_2_, 11-keto-17β-estradiol; 11KT, 11-ketotestosterone; 11OHA4, 11β-hydroxyandrostenedione; 11OHE_1_, 11β-hydroxyestrone; 11OHE_2_, 11β-hydroxy-17β-estradiol; 11OHT, 11β-hydroxytestosterone; A4, androstenedione; BPA, bisphenol A; CE, collision energy; CV, cone voltage; D2-T, testosterone 1,2-D2; D7-11OHA4, 11β-hydroxyandrostenedione 2,2,4,6,6,16,16-D7; E_1_, estrone; E_2_, 17β-estradiol; eV, electron volt; MRM, multiple reaction monitoring; m/z, mass-to-charge ratio; RT, retention time; T, testosterone.

### Whole-cell binding assay

Competitive whole-cell binding assays were performed in the COS-1 cell line as previously described (18). *K*_i_ ± SEM values for 11β-hydroxy-17β-estradiol (11OHE_2_) were determined from heterologous displacement curves using the median effective concentration (EC_50_) value, the published *K*_d_ values for E_2_ (18), and the concentration of radiolabeled E_2_, according to the equation by Cheng and Prusoff (19).

### Luciferase-reporter assays

Luciferase-reporter assays for estrogen receptor α (ERα) and ERβ were performed in HEK293 cells as previously described (18).

### Real-time proliferation assay

Real-time proliferation assays were carried out using an xCELLigence instrument (ACEA Biosciences). MCF7-BUS cells were maintained in phenol red-free DMEM supplemented with 5% charcoal-stripped FBS and 1% penicillin-streptomycin (starvation medium) for 1 week before the proliferation assay. At the start of the assay, 50-µL starved medium was added to each well of an E-plate 16 (ACEA Biosciences) and left to equilibrate for 30 minutes before taking a background reading. During this time a suspension of the MCF7-BUS cells (100 000 cells/mL) was prepared in starvation medium. A total of 100 µL of the cell suspension was then added to each well and the plate was equilibrated for a further 30 minutes prior to initializing the run on the xCELLigence instrument. Each run consisted of 400 sweeps with a 30-minute time interval. After 24 hours, 50 µL of unsupplemented phenol red-free DMEM containing 1 nM steroid (E_2_ or 11OHE_2_) was added to each well and proliferation monitored over 140 hours.

### BrdU-based cell proliferation assay

MCF7arom cells were starved with phenol red-free and serum-free medium for 24 hours before treatment with the indicated androgen and drug treatments. Cell growth was subsequently measured using the BrdU cell proliferation assay (Roche Diagnostics). Anti-BrdU–peroxidase immune complexes were detected by substrate reaction and quantified in an enzyme-linked immunosorbent assay reader at 370 nm.

### Analysis of relative gene expression by quantitative polymerase chain reaction

RNA extraction and complementary DNA synthesis were performed with TriReagent (Sigma-Aldrich) and the GoScript Reverse Transcription System kit (Promega). Quantitative PCR (qPCR) analysis was performed using the KAPA SYBR FAST qPCR Master Mix for LightCycler with a LightCycler96 rapid thermal cycler instrument (Roche Life Science). Primer sequences were as follows: estrogen-responsive pS2 gene (pS2) forward 5′-ATACCATCGACGTCCCTCCA-3′, pS2 reverse 5′-AAGCGTGTCTGAGGTGTCCG-3′, cathepsin D (CTSD) forward 5′-GCGAGTACATGATCCCCTGT-3′, CTSD reverse 5′-CTCTGGGGACAGCTTGTAGC-3′, progesterone receptor (PR) forward 5′-CTTAATCAACTAGGCGAGAG-3′, PR reverse 5′-AAGCTCATCCAAGAATACTG-3′ glyceraldehyde-3-phosphate dehydrogenase (GAPDH) forward 5′-TGAACGGGAAGCTCACTGG-3′, GAPDH reverse 5′-TCCACCACCCTGTTGCTGTA-3′. Transcript levels were calculated relative to GAPDH transcript levels using the method described by Pfaffl (20).

### Statistical analysis

All statistical analyses were performed in GraphPad Prism (version 9). Paired *t* tests (2-tailed) were used to compare Log *K*_d_/*K*_i_ values of E_2_ and 11OHE_2_ for ERα and ERβ. Unpaired *t* tests (2-tailed) were used to compare the substrate utilization of each steroid to its substrate-specific control. For all other experiments statistical significance was determined by comparing each treatment to the vehicle control using a one-way analysis of variance and Dunnett’s multiple comparisons test.

## Results

### 11-oxygenated androgens are aromatizable in vitro

**Figure 1.**
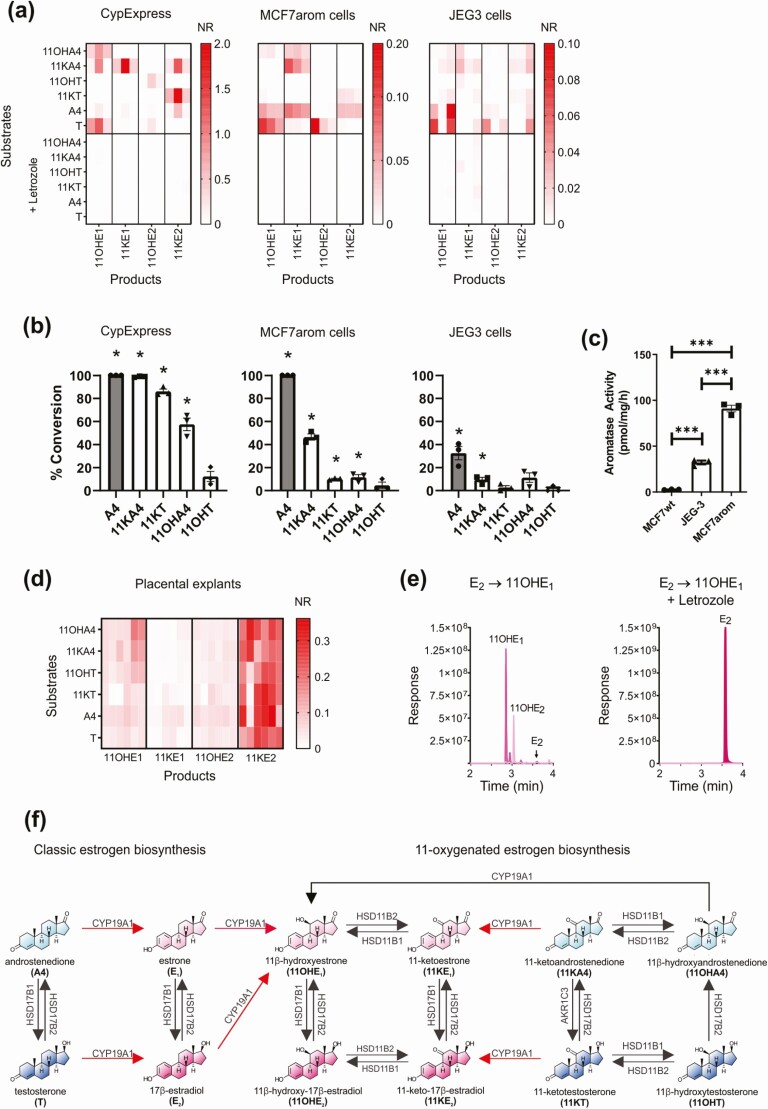
Biosynthesis of 11-oxygenated estrogens from 11-oxygenated and classic androgens. A, Biosynthesis of 11-oxygenated estrogens by 3 in vitro aromatase expression systems. Assays (n = 3) were performed using 1-µM androgen substrate with and without 10-µM letrozole. Owing to the lack of commercially available 11-oxygenated estrogen standards, conversion data are expressed as the mean normalized responses (NR) from independent experiments each performed in triplicate (NR: [peak area/peak area of the internal standard]/protein). B, Percentage conversion of aromatase substrates as calculated substrate utilization. Data points are shown as mean ± SEM from 3 independent experiments each performed in triplicate. Statistical analysis was performed by comparing each steroid to its substrate specific control. *P less than .05; unpaired *t* test. C, Aromatase activity in MCF7 (wild type), JEG3, and MCF7arom cells was determined using a tritiated water-release assay using A4 (1β-3H[N]) as substrate. Data represent 3 independent experiments shown as mean ± SEM. ***P less than .001; one-way analysis of variance and Dunnett’s multiple comparisons test. D, Biosynthesis of 11-oxygenated estrogens by human placenta explants (n = 6). E, Representative (multiple reaction monitoring [MRM]) chromatograms demonstrating the aromatase-catalyzed conversion of 1-µM E_2_ directly to 11OHE_1_ in the CypExpress system (n = 3). Conversion was inhibited by the addition of 10-µM letrozole. F, Schematic overview of 11-oxygenated estrogen biosynthesis. Androgens and estrogens are shown in blue and pink, respectively. All observed aromatase catalyzed reactions are shown in red. Steroids: 11OHA4, 11β-hydroxyandrostenedione; 11KA4, 11-ketoandrostenedione; 11OHT, 11β-hydroxytestosterone; 11KT, 11-ketotestosterone; 11OHE_1_, 11β-hydroxyestrone; 11OHE_2_, 11β-hydroxy-17β-estradiol; 11KE_1_, 11-ketoestrone; 11KE_2_, 11-keto-17β-estradiol; A4, androstenedione; E_1_, estrone; E_2_, 17β-estradiol; T, testosterone.

Using a combination of in vitro and ex vivo test systems, and employing liquid chromatography–tandem mass spectrometry (LC-MS/MS) for steroid analysis, we tested whether 11-oxygenated androgens are substrates for human aromatase. The in vitro assays comprised 3 cell-based test systems expressing aromatase: a reconstituted lysate from yeast overexpressing human aromatase (CypExpress); an MCF7 human breast cancer cell line engineered to overexpress aromatase (MCF7arom) (12); and a human placental cell line, which endogenously expresses aromatase (JEG3). In all 3 aromatase-expression systems, 11-oxygenated estrogens were detected after incubations with 11OHA4, 11KA4, and 11KT (Fig. 1A). These included 11OHE_1_, 11OHE_2_, 11KE_1_, and 11KE_2_. We did not detect 11-oxygenated estrogens after incubation with 11OHT. The addition of the specific aromatase inhibitor, letrozole, abolished all observed conversions (Fig. 1A). Analysis of substrate utilization revealed that 11-oxygenated androgens were not all aromatized to the same extent, with significant conversion for 11KA4 observed in all 3 cell-based test systems and for 11KT and 11OHA4 in 2 of the 3 systems, CypExpress and MCF7arom cells (Fig. 1B). Substrate utilization was abolished by the addition of letrozole, thereby suggesting that the substrates were converted by aromatase only (see Fig. 1B). All 3 cell-based systems catalyzed the aromatization of the classic aromatase substrates, A4 and T (see Fig. 1B), though conversion was markedly lower in JEG3 cells, which had lower aromatase activity than the MCF7arom cells (Fig. 1C).

Next, we analyzed the aromatase-mediated biosynthesis of 11-oxygenated estrogens using explants obtained from fresh human placenta. In this ex vivo assay, LC-MS/MS detected 11-oxygenated estrogens after incubation with each of the 4 11-oxygenated androgens (Fig. 1D).

### Aromatase has 11β-hydroxylase activity toward classic estrogens

When we used the classic aromatase substrates, A4 and T, we observed the production of 11-oxygenated estrogens in all in vitro aromatase test systems and in human placenta explants (Fig. 1A and 1D), suggesting that aromatase itself might exert 11β-hydroxylase activity. Incubations of the CypExpress aromatase expression system with T (see Fig. 1A), E_2_ (Fig. 1E), or E_1_ (data not shown) yielded the 11-oxygenated estrogen, 11OHE_1_; these conversions were abolished by the addition of letrozole. Contamination with an 11β-hydroxylase can be eliminated as neither A4 nor T was converted to 11OHA4 or 11OHT, as would have been expected in the presence of CYP11B1 or CYP11B2. Moreover, incubations of the human 11β-hydroxylase enzymes CYP11B1 or CYP11B2 with E_2_ or E_1_ failed to yield 11-oxygenated estrogens (data not shown). This is in agreement with a previous study that failed to detect the 11β-hydroxylation of E_1_ by human adrenal slices (21).

### 11-oxygenated estrogens are bona fide estrogens

**Figure 2.**
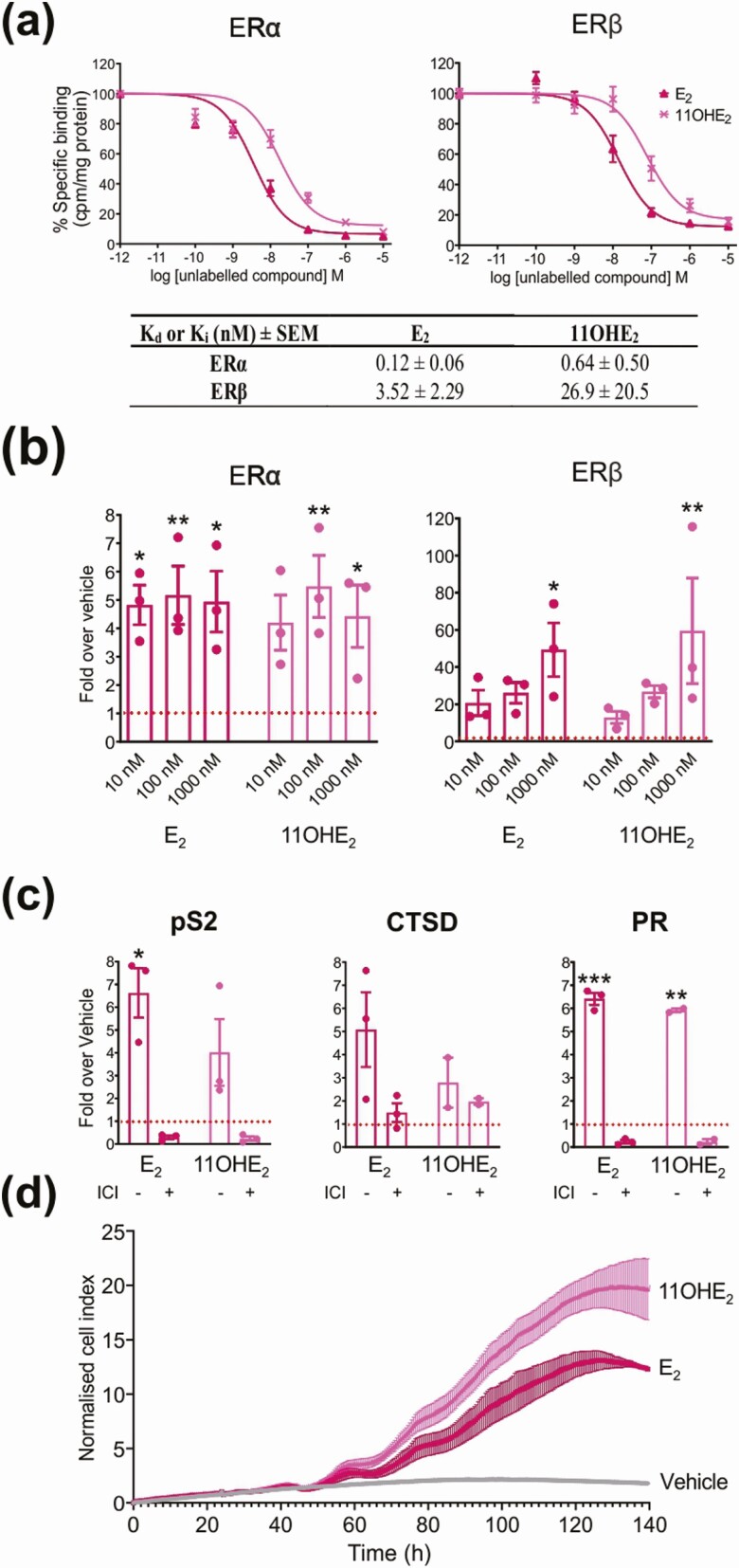
11-Oxygenated estrogens bind and activate the human estrogen receptors α and β. A, Binding affinities of 11β-hydroxy-17β-estradiol (11OHE_2_) relative to 17β-estradiol (E_2_) as determined by whole-cell binding assays (n = 3). Binding data were analyzed with nonlinear regression assuming competitive binding to one class of binding sites, and the *K*_i_ ± SEM values for 11OHE_2_ determined from a heterologous displacement curve. B, Transactivation of human estrogen receptor α (Erα) and ERβ by 11OHE_2_ and E_2_ (n = 3). *P less than .05; **P less than .005; one-way analysis of variance (ANOVA) and Dunnett’s multiple comparisons test, compared to the vehicle control set as 1. C, Induction of ER-regulated gene expression (pS2, estrogen-responsive pS2 gene; CTSD, cathepsin D; PR, progesterone receptor) in MCF7-BUS cells by 1-nM 11OHE_2_ and E_2_ in the absence and presence of 1-µM fulvestrant (ICI). *P less than .05; **P less than .005; and ***P less than .001; one-way ANOVA and Dunnett’s multiple comparisons test, compared to the vehicle control set as 1. D, Real-time proliferation of MCF7-BUS cells as induced by 1-nM 11OHE_2_ and E_2_ (n = 3). All results are represented as mean ± SEM of 3 independent experiments, each performed in triplicate.

After confirming the biosynthesis of 11-oxygenated estrogens by human aromatase in vitro and ex vivo, we next set out to determine their estrogenic activity. First, we confirmed that 11OHE_2_, the only commercially available 11-oxygenated estrogen, binds to both subtypes of the nuclear human ER, ERα and ERβ. The dissociation equilibrium constants (*K*_i_) were 0.64 and 26.9 nM for ERα and ERβ, respectively (Fig. 2A). Next, using a promoter-reporter system, we showed that 11OHE_2_ could transactivate via both ERα and ERβ to a similar degree (not significantly different) to E_2_ (Fig. 2B). Cells transfected only with the reporter showed no response (data not shown), thereby confirming activity via ERα or ERβ. These results were confirmed by demonstrating that the 11OHE_2_-induced expression of endogenous estrogen-responsive genes in cells expressing both ER subtypes was abolished by ICI, a selective ER downregulator (Fig. 2C) (22). The estrogenic properties of 11OHE_2_ were further confirmed by demonstrating that 11OHE_2_, like E_2_, induced significant cell growth (*P* = .003) in the estrogen-responsive MCF7-BUS breast cancer cell line (Fig. 2D). Our findings are in agreement with a previous study that showed that 11OHE_2_ can bind to the ER and induce proliferation in estrogen-responsive MCF-7 breast cancer cells (23).

### Aromatization of 11-ketotestosterone yields an estrogenic response

**Figure 3.**
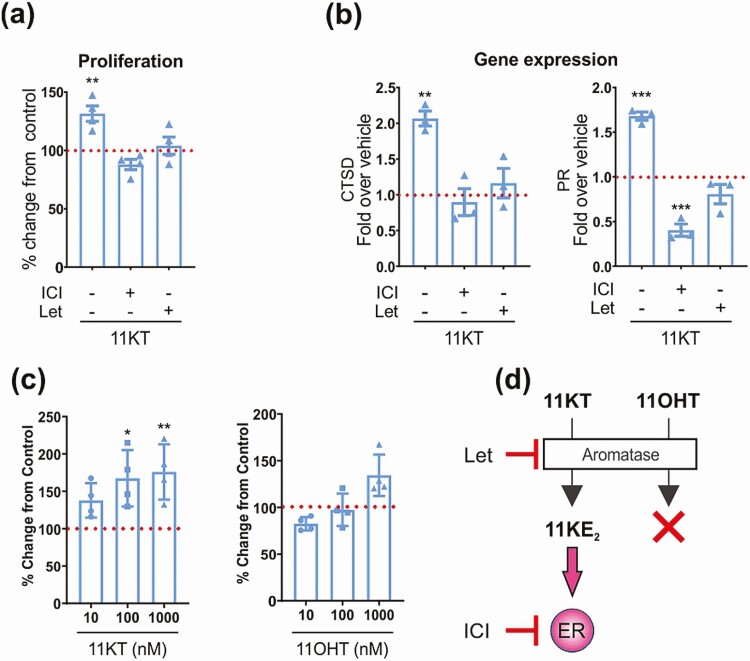
Aromatization of 11-ketotestosterone (11KT) elicits an estrogen response. A, 11KT-mediated estrogen-dependent proliferation (100 nM) as measured by a BrdU proliferation assay is inhibited by 1-µM letrozole or fulvestrant in MCF7arom cells (n = 3). **P less than .005; one-way analysis of variance (ANOVA) and Dunnett’s multiple comparisons test, compared to the control set as 100%. B, 11KT-mediated (100 nM) increase in estrogen-dependent gene expression (cathepsin D [CTSD] and progesterone receptor [PR]) is inhibited by 1-µM letrozole or fulvestrant in MCF7arom cells (n = 3). **P less than .005 and ***P less than .001; one-way ANOVA and Dunnett’s multiple comparisons test, compared to the vehicle control set as 1. C, 11KT, but not 11-hydroxytestosterone (11OHT), induce estrogen-dependent proliferation in MCF7arom as measured by a BrdU proliferation assay. *P less than .05 and **P less than .005; one-way ANOVA and Dunnett’s multiple comparisons test, compared to control set as 100%. D, Schematic illustrating the aromatase and ER-dependent estrogen response elicited by 11KT, but not 11OHT. Experiments A, B, and C are represented as mean ± SEM of A and B, 3, or C, 4 independent experiments, each performed in triplicate.

Next, we determined whether the aromatization of the potent androgen 11KT could result in ER-mediated effects. We found that the aromatization of 11KT yielded the bioactive 11-oxygenated estrogen 11KE_2_ (see Fig. 1A and 1D), which induced cell growth (Fig. 3A) and an increase in estrogen-dependent gene expression (Fig. 3B) in MCF7arom cells. These effects were abolished by the addition of either letrozole or ICI, thereby confirming that 11KT-mediated estrogen responses require both functional aromatase and ER (Fig. 3). As anticipated, 11OHT, which we demonstrated not to be aromatizable (see Fig. 1A), failed to yield an estrogenic response (Fig. 3C and 3D).

### 11-oxygenated estrogens are not detectable in circulation

Finally, after demonstrating that 11-oxygenated androgens are aromatizable in vitro and ex vivo, we used LC-MS/MS to screen for the presence of 11-oxygenated estrogens in human serum. We collected maternal serum and umbilical cord blood at birth in 6 normal pregnancies, chosen because of the high levels of aromatase expressed during pregnancy. In addition, we analyzed serum from 10 patients with increased circulating 11-oxygenated androgen concentrations due to congenital adrenal hyperplasia, that is, increased substrate availability for 11-oxygenated estrogen biosynthesis. We also analyzed serum samples from 7 patients with steroidogenically active adrenocortical carcinoma and increased 11-oxygenated adrenal androgen excretion, that is, increased substrate availability and potentially dysregulated steroidogenesis due to ectopic adrenal aromatase expression. In all samples we detected classic estrogens, but no 11-oxygenated estrogens (data not shown). Our limit of detection for 11OHE2, the only commercially available 11-oxygenated estrogen, was 20 pM and concentrations lower than this could therefore have gone undetected. Limits of detection could not be determined for the other 11-oxygenated estrogens because of a lack of standards.

## Discussion

Despite being overlooked for several decades, it is now clear that 11-oxygenated androgens make a substantial contribution to the circulating androgen pool, particularly in women (1-5). Because androgens are the obligatory precursors to estrogen biosynthesis, it is essential to consider if 11-oxygenated androgens are aromatizable or if they serve as nonaromatizable androgens. This is the first study to describe the aromatization of 11-oxygenated androgens in vitro. We show that 11KA4, 11OHA4, and 11KT are all aromatizable, albeit to a lower degree than the classic aromatase substrates. 11OHT was the only nonaromatizable 11-oxygenated androgen in the 3 cell-based aromatase expression systems employed in this study. The detection of 11-oxygenated estrogens in the human placenta explant culture incubations with 11OHT can be explained by the prevalent expression of HSD11B2 and HSD17B2 in placental tissue (24). These enzymes can convert 11OHT to 11KT and 11OHA4, respectively (1) (Fig. 1F), which both were aromatized in all in vitro and ex vivo systems we employed. Similarly, the accumulation of 11KE_2_ as the primary 11-oxygenated estrogen product in the placenta explant cultures is explained by the high expression of HSD11B2 and the estrogen-converting enzyme HSD17B1 in human placenta (24) (Fig. 1F).

Our data also revealed that aromatase can catalyze the 11β-hydroxylation of estrogens resulting from the aromatization of A4 and T. This activity has likely previously gone undetected because of the longstanding use of tritiated water-release assays for aromatase activity that do not directly identify the measured estrogen products (25-27). Dual aromatase and 11β-hydroxylase activity catalyzed by a single enzyme is supported by the observation that CYP11B has a secondary aromatase function (28, 29). Incubations of aromatase with either T or E_2_ yielded primarily 11OHE_1_ and not 11OHE_2_ as one would anticipate, suggesting additional 17β-hydroxysteroid dehydrogenase activity (Fig. 1E and 1F), which warrants further investigation in the future. Incubations of the human 11β-hydroxylase enzymes CYP11B1 or CYP11B2 with E_2_ or E_1_ failed to yield 11-oxygenated estrogens (data not shown), demonstrating that although these enzymes are essential for the biosynthesis of 11-oxygenated androgens (30, 31), they play no direct role in the production of 11-oxygenated estrogens. In agreement with our findings, a previous study had shown that incubations of human adrenal slices, the exclusive site of CYP11B1 and CYP11B2 expression, failed to yield 11β-hydroxylated products when using E_1_ as a substrate (21).

In terms of activity, we show that 11OHE_2_, the only commercially available 11-oxygenated estrogen, binds and activates ERα and ERβ in a similar manner to E_2_. 11OHE_2_ also stimulated proliferation and expression of estrogen-responsive genes in an estrogen-responsive breast cancer cell line, thereby confirming its estrogenic activity. Moreover, aromatization of the potent 11-oxygenated androgen, 11KT, to 11KE_2_ in MCF7arom cells (see Fig. 1A) induced cell growth (Fig. 3A) and an increase in estrogen-dependent gene expression (Fig. 3B), thereby indirectly demonstrating the 11KE_2_ is also a bona fide estrogen. The relative estrogenic activity of 11OHE_2_ and 11KE_2_ can be determined only when 11KE_2_ becomes commercially available or is custom synthesized. This comparison will be of interest given that 11KT is a more efficacious androgen than 11OHT (1). It also remains to be determined whether 11OHE_1_ and 11KE_1_ are estrogenic. Our results differ from one previous study conducted in wild-type MCF7 cells that concluded that 11KT is not aromatizable (23). However, this is likely explained by the much lower aromatase activity in wild-type MCF7 cells as compared to the aromatase-overexpressing MCF7arom cell line we used for our experiments (see Fig. 1C).

Indeed, the degree of aromatization observed was dependent on the level of aromatase expression and activity in our in vitro test systems, with only 11KA4 being significantly aromatized in JEG3 cells, which had the lowest aromatase activity (Fig. 1B and 1C). Even in MCF7arom cells, only 46% of 11KA4, the best 11-oxygenated androgen substrate, was aromatized, whereas 100% of A4 was converted to E_1_. The comparatively poor aromatization of 11-oxygenated androgens was further highlighted by the inability to detect 11-oxygenated estrogens in serum samples representative of high aromatase expression (pregnancy and cord serum) or high substrate levels (congenital adrenal hyperplasia or adrenocortical carcinoma with elevated 11-oxygenated androgen levels). These findings suggest that while 11-oxygenated androgens are aromatizable in vitro and even ex vivo, aromatization may be limited in vivo because of the relatively low activity of aromatase toward 11-oxygenated androgens in comparison to the classic substrates. The 11-oxygenated androgens may therefore serve almost exclusively as androgens in vivo and not also as estrogen precursors. However, the intracrine biosynthesis of 11-oxygenated estrogens within aromatase-expressing peripheral target cells cannot be ruled out. Once standards for the 11-oxygenated estrogens become available, this will allow for the more comprehensive quantification of these estrogens in serum and tissue. Interestingly, using paper chromatography, and chemical and infrared analyses, 11OHE_1_ was previously reported to be a major product of a feminizing adrenal carcinoma (32), suggesting that in vivo biosynthesis of 11-oxygenated estrogens does occur under certain circumstances.

## Data Availability

Some or all datasets generated during and/or analyzed during the current study are not publicly available but are available from the corresponding author on reasonable request.

## Abbreviations

11KA4: 11-ketoandrostenedione
11KE_1_: 11-ketoestrone
11KE_2_: 11-keto-17β-estradiol
11KT: 11-ketotestosterone
11OHA4: 11β-hydroxyandrostenedione
11OHE_1_: 11β-hydroxyestron
11OHE_2_: 11β-hydroxy-17β-estradiol
A4: androstenedione
AR: androgen receptor
BPA: bisphenol A
CYP11B1: cytochrome P450 11β-hydroxylase
D2-T: testosterone 1,2-D2
D7-11OHA4: 11β-hydroxyandrostenedione 2,2,4,6,6,16,16-D7
DHT: 5α-dihydrotestosterone
DMEM: Dulbecco’s Modified Eagle Medium
E_1_: estrone
E_2_: 17β-estradiol
ER: estrogen receptor
FBS: fetal bovine serum
HSD11B2: 11β-hydroxysteroid dehydrogenase type 2
HSD17B2: 17β-hydroxysteroid dehydrogenase type 2
ICI: fulvestrant
LC-MS/MS: liquid chromatography–tandem mass spectrometry
MRM: multiple reaction monitoring
PR: progesterone receptor
qPCR: quantitative polymerase chain reaction
T: testosterone

## Additional Information

***Disclosures:*** The authors have nothing to disclose.

## References

[1] Storbeck KH, Bloem LM, Africander D, Schloms L, Swart P, Swart AC. 11β-Hydroxydihydrotestosterone and 11-ketodihydrotestosterone, novel C19 steroids with androgenic activity: a putative role in castration resistant prostate cancer? Mol Cell Endocrinol. 2013;377(1-2):135-146. 23856005 10.1016/j.mce.2013.07.006

[2] Rege J, Nakamura Y, Satoh F, et al Liquid chromatography–tandem mass spectrometry analysis of human adrenal vein 19-carbon steroids before and after ACTH stimulation. J Clin Endocrinol Metab. 2013;98(3):1182-1188. 23386646 10.1210/jc.2012-2912 PMC3590473

[3] Pretorius E, Arlt W, Storbeck KH. A new dawn for androgens: novel lessons from 11-oxygenated C19 steroids. Mol Cell Endocrinol. 2017;441:76-85. 27519632 10.1016/j.mce.2016.08.014

[4] Turcu AF, Auchus RJ. Clinical significance of 11-oxygenated androgens. Curr Opin Endocrinol Diabetes Obes. 2017;24(3):252-259. 28234803 10.1097/MED.0000000000000334 PMC5819755

[5] Barnard M, Quanson JL, Mostaghel E, Pretorius E, Snoep JL, Storbeck KH. 11-Oxygenated androgen precursors are the preferred substrates for aldo-keto reductase 1C3 (AKR1C3): implications for castration resistant prostate cancer. J Steroid Biochem Mol Biol. 2018;183:192-201. 29936123 10.1016/j.jsbmb.2018.06.013 PMC6283102

[6] Pretorius E, Africander DJ, Vlok M, Perkins MS, Quanson J, Storbeck KH. 11-Ketotestosterone and 11-ketodihydrotestosterone in castration resistant prostate cancer: potent androgens which can no longer be ignored. PLoS One. 2016;11(7):e0159867. 27442248 10.1371/journal.pone.0159867 PMC4956299

[7] O’Reilly MW, Kempegowda P, Jenkinson C, et al. 11-Oxygenated C19 steroids are the predominant androgens in polycystic ovary syndrome. J Clin Endocrinol Metab. 2017;102(3):840-848. 27901631 10.1210/jc.2016-3285 PMC5460696

[8] Nanba AT, Rege J, Ren J, Auchus RJ, Rainey WE, Turcu AF. 11-Oxygenated C19 steroids do not decline with age in women. J Clin Endocrinol Metab. 2019;104(7):2615-2622. 30753518 10.1210/jc.2018-02527 PMC6525564

[9] Turcu AF, Nanba AT, Chomic R, et al. Adrenal-derived 11-oxygenated 19-carbon steroids are the dominant androgens in classic 21-hydroxylase deficiency. Eur J Endocrinol. 2016;174(5):601-609. 26865584 10.1530/EJE-15-1181 PMC4874183

[10] Skiba MA, Bell RJ, Islam RM, Handelsman DJ, Desai R, Davis SR. Androgens during the reproductive years: what is normal for women? J Clin Endocrinol Metab. 2019;104(11):5382-5392. 31390028 10.1210/jc.2019-01357

[11] Davio A, Woolcock H, Nanba AT, et al Sex differences in 11-oxygenated androgen patterns across adulthood. J Clin Endocrinol Metab. 2020;105(8):e2921-e2929. 10.1210/clinem/dgaa343 PMC7340191 32498089

[12] Zhou DJ, Pompon D, Chen SA. Stable expression of human aromatase complementary DNA in mammalian cells: a useful system for aromatase inhibitor screening. Cancer Res. 1990;50(21):6949-6954. 2208160

[13] Barnard L, Gent R, van Rooyen D, Swart AC. Adrenal C11-oxy C_21_ steroids contribute to the C11-oxy C_19_ steroid pool via the backdoor pathway in the biosynthesis and metabolism of 21-deoxycortisol and 21-deoxycortisone. J Steroid Biochem Mol Biol. 2017;174:86-95. 28774496 10.1016/j.jsbmb.2017.07.034

[14] Quanson JL, Stander MA, Pretorius E, Jenkinson C, Taylor AE, Storbeck KH. High-throughput analysis of 19 endogenous androgenic steroids by ultra-performance convergence chromatography tandem mass spectrometry. J Chromatogr B Analyt Technol Biomed Life Sci. 2016;1031: 131-138. 10.1016/j.jchromb.2016.07.024 27479683

[15] Anari MR, Bakhtiar R, Zhu B, Huskey S, Franklin RB, Evans DC. Derivatization of ethinylestradiol with dansyl chloride to enhance electrospray ionization: application in trace analysis of ethinylestradiol in rhesus monkey plasma. Anal Chem. 2002;74(16):4136-4144. 12199585 10.1021/ac025712h

[16] Nelson RE, Grebe SK, O’Kane DJ, Singh RJ. Liquid chromatography–tandem mass spectrometry assay for simultaneous measurement of estradiol and estrone in human plasma. Clin Chem. 2004;50(2):373-384. 14656902 10.1373/clinchem.2003.025478

[17] Kushnir MM, Rockwood AL, Yue B, Meikle AW. High sensitivity measurement of estrone and estradiol in serum and plasma using LC-MS/MS. In: Garg U, Hammett-Stabler CA, eds. Methods in Molecular Biology.Vol. 603. Humana Press; 2010:219- 228. 10.1007/978-1-60761-459-3_20 20077073

[18] Perkins MS, Louw-du Toit R, Africander D. A comparative characterization of estrogens used in hormone therapy via estrogen receptor (ER)-α and -β. J Steroid Biochem Mol Biol. 2017;174:27-39. 28743541 10.1016/j.jsbmb.2017.07.022

[19] Cheng Y-C, Prusoff WH. Relationship between the inhibition constant (KI) and the concentration of inhibitor which causes 50 per cent inhibition (I50) of an enzymatic reaction. Biochem Pharmacol. 1973;22(23):3099-3108. 4202581 10.1016/0006-2952(73)90196-2

[20] Pfaffl MW . A new mathematical model for relative quantification in real-time RT-PCR. Nucleic Acids Res. 2001; 29(9):e45. 11328886 10.1093/nar/29.9.e45 PMC55695

[21] Knuppen R, Breuer H. Biogenesis of 11β-hydroxyoestrone and 16α-hydroxyoestrone by adrenal tissue. Biochim Biophys Acta. 1962;58:147-148. 14457398 10.1016/0006-3002(62)90834-x

[22] Lai AC, Crews CM. Induced protein degradation: an emerging drug discovery paradigm. Nat Rev Drug Discov. 2017;16(2):101-114. 27885283 10.1038/nrd.2016.211 PMC5684876

[23] Wiese TE, Polin LA, Palomino E, Brooks SC. Induction of the estrogen specific mitogenic response of MCF-7 cells by selected analogues of estradiol-17β: A 3D QSAR study. J Med Chem. 1997;40(22):3659-3669. 9357533 10.1021/jm9703294

[24] Ryan KJ . Biological aromatization of steroids. J Biol Chem. 1959;234(2):268-272. 13630892

[25] Rabe T, Rabe D, Runnebaum B. New aromatase assay and its application for inhibitory studies of aminoglutethimide on microsomes of human term placenta. J Steroid Biochem. 1982;17(3):305-309. 7132348 10.1016/0022-4731(82)90204-7

[26] Tilson-Mallett N, Santner SJ, Feil PD, Santen RJ. Biological significance of aromatase activity in human breast tumors. J Clin Endocrinol Metab. 1983;57(6):1125-1128. 6630410 10.1210/jcem-57-6-1125

[27] Lephart ED, Simpson ER. Assay of aromatase activity. Methods Enzymol. 1991;206:477-483. 1784232 10.1016/0076-6879(91)06116-k

[28] Suhara K, Ohashi K, Takeda K, Katagiri M. P-450_11β_-dependent conversion of androgen to estrogen, the aromatase reaction. Biochem Biophys Res Commun. 1986;140(2):530-535. 3490848 10.1016/0006-291x(86)90764-3

[29] Suhara K, Ohashi K, Takahashi K, Katagiri M. Aromatase and nonaromatizing 10-demethylase activity of adrenal cortex mitochondrial P-450_11β_. Arch Biochem Biophys. 1988;267(1):31-37. 3264134 10.1016/0003-9861(88)90004-5

[30] .Schloms L, Storbeck KH, Swart P, Gelderblom WC, Swart AC. The influence of *Aspalathus linearis* (Rooibos) and dihydrochalcones on adrenal steroidogenesis: quantification of steroid intermediates and end products in H295R cells. J Steroid Biochem Mol Biol. 2012;128(3-5):128-138. 22101210 10.1016/j.jsbmb.2011.11.003

[31] Swart AC, Schloms L, Storbeck K-H, et al 11β-Hydroxyandrostenedione, the product of cytochrome P450 11β-hydroxylase: a novel substrate for 11β-hydroxysteroid dehydrogenase and 5α-reductase. J Steroid Biochem Mol Biol. 2013;138:132-142. 23685396 10.1016/j.jsbmb.2013.04.010

[32] Mahesh VB, Herrmann W. Isolation of estrone and 11β-hydroxy estrone from a feminizing adrenal carcinoma. Steroids. 1963;1(1):51-61.

